# Pneumomediastinum in a patient with COVID-19 due to diffuse alveolar damage

**DOI:** 10.1136/bcr-2021-242527

**Published:** 2021-05-11

**Authors:** Frank Heijboer, Laurien Oswald, Sander Cretier, Gert-Jan Braunstahl

**Affiliations:** 1 Pulmonology, Franciscus Gasthuis en Vlietland, Rotterdam, The Netherlands; 2 Radiology, Franciscus Gasthuis en Vlietland, Rotterdam, The Netherlands; 3 Pulmonology, Erasmus Medical Center, Rotterdam, The Netherlands

**Keywords:** COVID-19, pneumomediastinum, air leaks

## Abstract

A 74-year-old man with COVID-19 was admitted and experienced progressive dyspnoea while receiving supplemental oxygen via high-flow nasal cannula (HFNC). A CT of the thorax showed a pneumomediastinum. The HFNC was temporally interrupted, since it was uncertain whether the positive end-expiratory pressure of the HFNC could be the cause of the pneumomediastinum. After restart of the HFNC, there was no increase of symptoms. We suggest that the pneumomediastinum was the result of COVID-19-related alveolar damage, and not due to the use of HFNC. This observation is relevant since HFNC is often used in the treatment of severe COVID-19 pneumonia.

## Background

Little is known about pneumomediastinum in patients with COVID-19 and whether high-flow nasal cannula (HFNC) can provoke or worsen a pneumomediastinum. Supplemental oxygen therapy via HFNC is of great importance in the treatment of hypoxemia in patients with COVID-19. However, HFNC may potentially generate some positive end-expiratory pressure by higher flow levels. High pressure in the airways puts stress on the airway wall and leads to small air leaks that may result in pneumothorax or pneumomediastinum. Therefore, it is key to assess if HFNC is harmful in COVID-19 patients with pneumomediastinum.

## Case presentation

A 74-year-old man, with no relevant comorbidities, presented to the emergency department with respiratory failure. For 7 days he had been suffering from fatigue, loss of appetite, coughing and progressive shortness of breath. At presentation he was confused, although there was no loss of orientation in space and time. He had no history of smoking or drug abuse. At admission, the patient was not suffering from chest pain.

Physical examination showed a man clearly short of breath with a respiratory rate of 51/min and an oxygen saturation (SO_2_) of 84% on room air. On a 15 L/min non-rebreathing mask (NRM) he improved to 100% SO_2_. He had a blood pressure of 137/78 mm Hg with a pulse of 139 beats per minute. His temperature was 36.3°C. Auscultation revealed bilateral fine crackles. Inspection and palpation of the skin did not reveal any subcutaneous crackling bubbles at that time. Also, there was no swelling of the neck.

**Figure 1:**
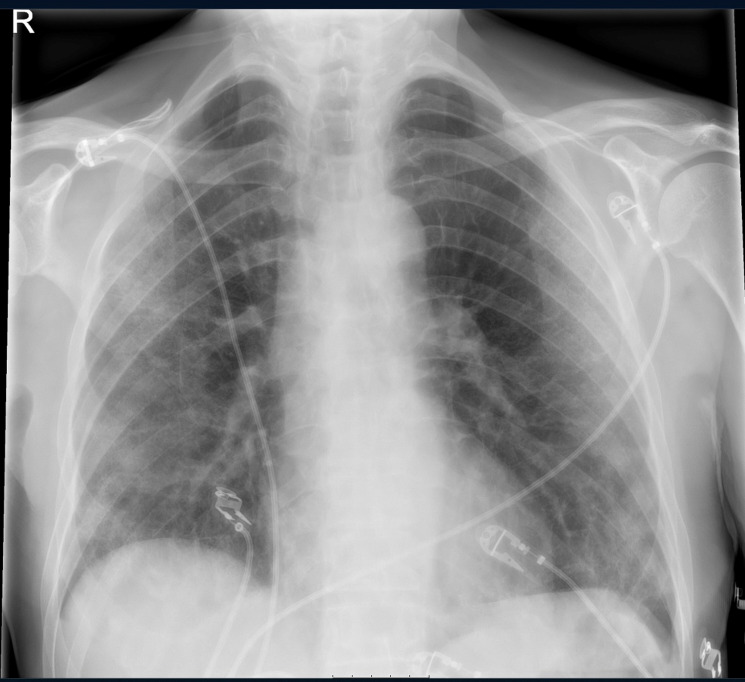
X-ray of the chest at admission, showing bilateral patchy infiltrates; no signs of pneumomediastinum of subcutaneous emphysema.

Bilateral patchy infiltrates were seen on his X-ray of the chest. There was no sign of a pneumomediastinum or subcutaneous emphysema at that time (figure 1). The patient’s C-reactive protein (CRP) was elevated at 245 mg/L (0–5 mg/L), as well as an elevated procalcitonin of 0.55 µg/L (0–0.50 µg/L). The polymerase chain reaction analysis for COVID-19 was positive.

The patient was started on dexamethasone 6 mg and prophylactic dalteparin 5000EH. Additionally, he received supplemental oxygen via HFNC (Optiflow) with settings flow 40 L and FiO_2_ 60%, which resulted in a respiratory rate of 28/min and an oxygen saturation of 96%.

On day 4, there was an increase in shortness of breath. The current settings of the HFNC were flow 40 L and FiO_2_ 70%; the FiO_2_ was raised over the previous days due to a persistently high respiratory rate and an oxygen saturation <94%. Another X-ray of the chest was made, and D-dimer was determined to find a cause for the increase in shortness of breath.

## Investigations

**Figure 2:**
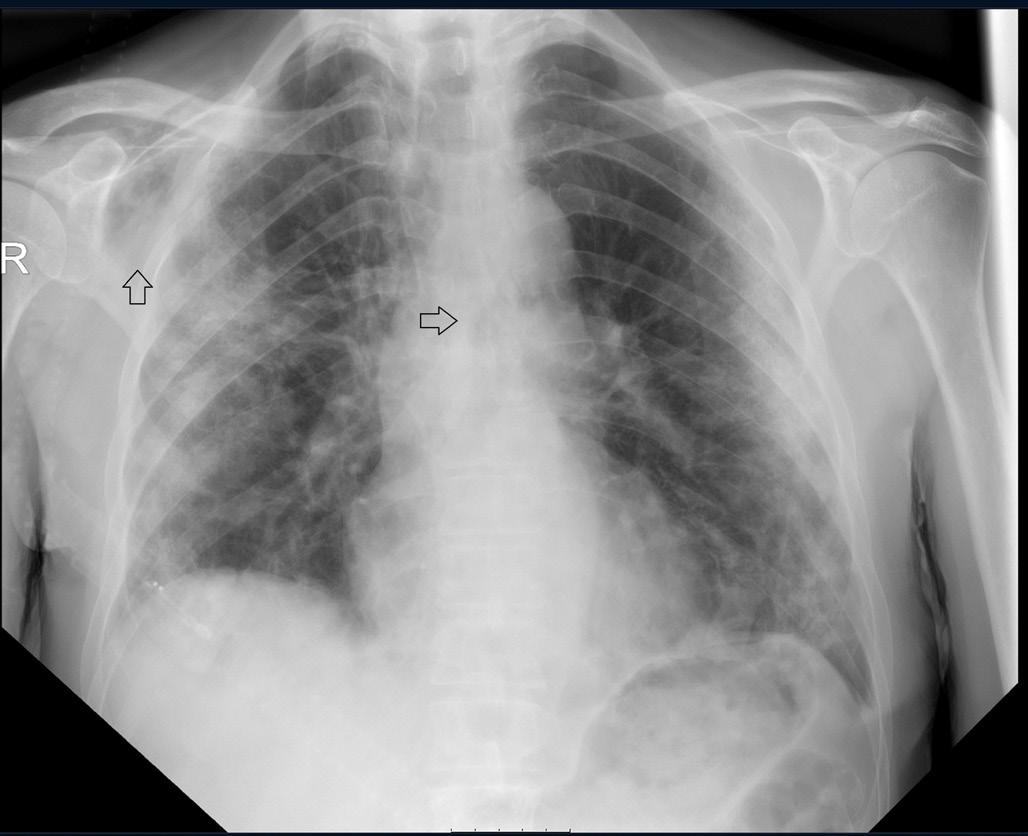
X-ray of the chest during the increase in shortness of breath, showing progressive bilateral patchy infiltrates; the arrow pointing at the right shoulder showing signs of subcutaneous emphysema; the arrow pointing at the mediastinum suggesting a pneumomediastinum.

**Figure 3:**
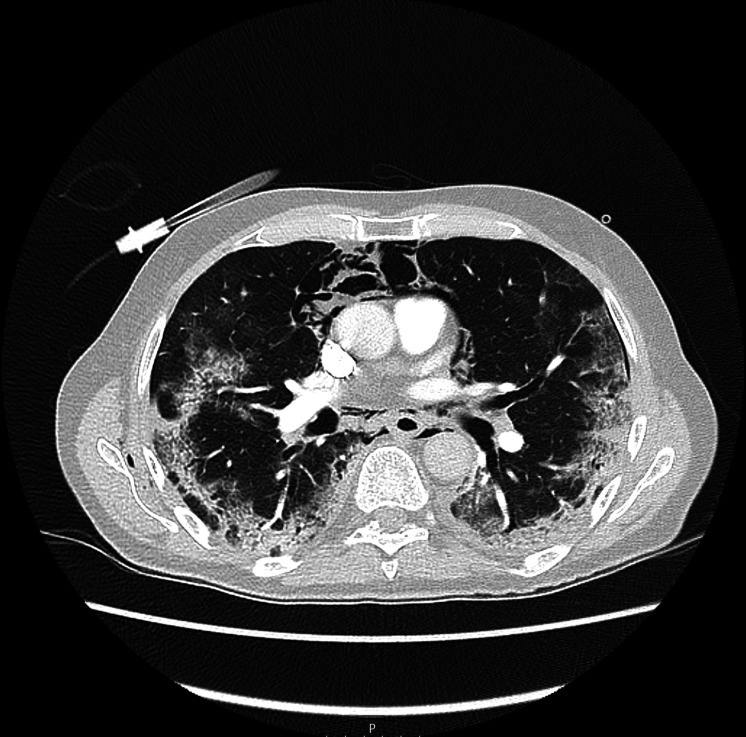
CT pulmonary angiogram (axial plane) during the increase of shortness of breath, showing a pneumomediastinum, subcutaneous emphysema and ground-glass opacities related to COVID-19.

**Figure 4:**
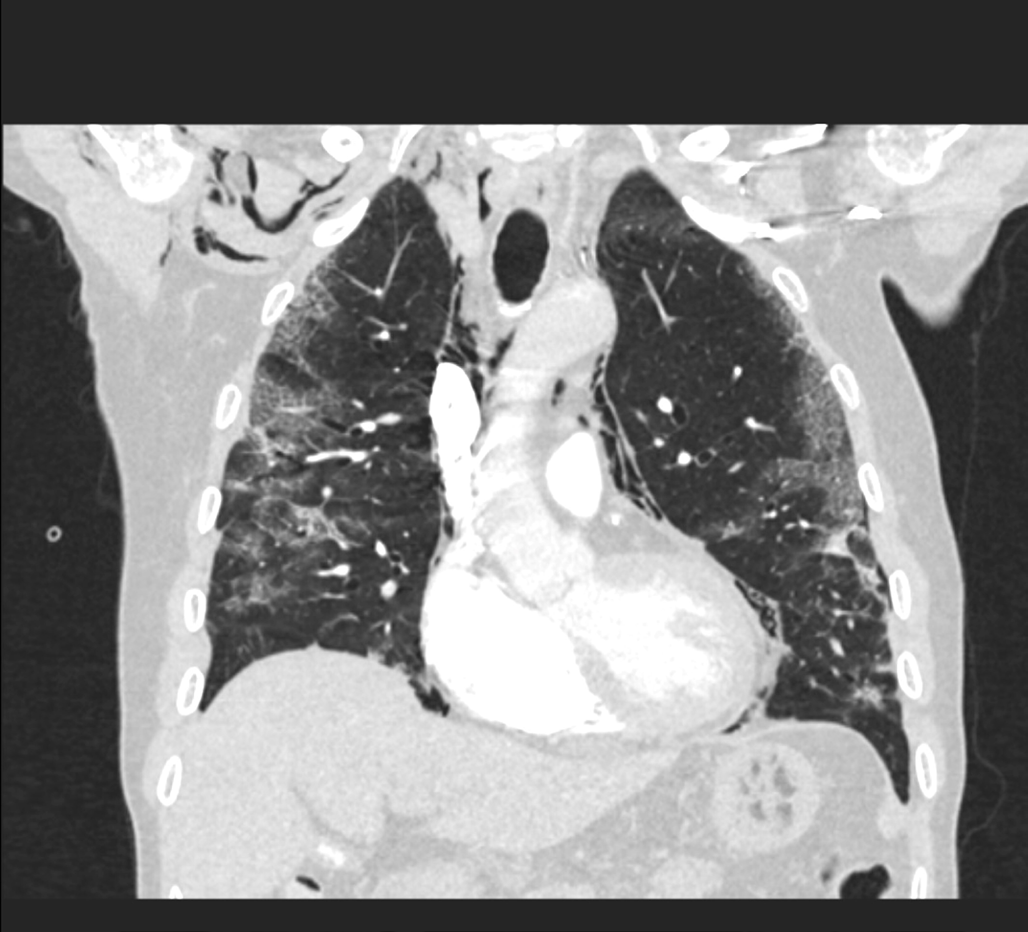
CT pulmonary angiogram (coronal plane) during the increase of shortness of breath, showing a pneumomediastinum, subcutaneous emphysema and ground-glass opacities related to COVID-19.

The X-ray of the chest showed an image with progressive bilateral patchy infiltrates compared with the previous X-ray of the chest with subcutaneous emphysema supraclavicular and near the scapula on the right side. No concomitant pneumothorax was seen on the X-ray of the chest (figure 2). The patient’s D-dimer was elevated at >9.00 mg/L (<0.50/L). A CT pulmonary angiogram was made, which excluded pulmonary embolism, but showed extensive pneumomediastinum, pneumopericardium and subcutaneous emphysema in the neck with a minimal pneumothorax laterally on the left (figures 3 and 4)

## Differential diagnosis

To find the cause of pneumomediastinum in this case, we had to differentiate between a rupture of the oesophagus or a pulmonary cause (eg, alveolar damage). The gastroenterologist was consulted to assess whether an oesophageal rupture could be the cause. This was ruled out, as the patient did not show any clinical symptoms nor radiological signs of a rupture or tracheal lesion. Moreover, the patient did not suffer from epigastric pain or dysphagia. No pleural of mediastinal fluid was seen on the CT pulmonary angiogram. In addition, no abnormalities of the oesophagus were spotted. The cause of the pneumomediastinum of the patient was seen as secondary to COVID-19 pneumonia.

## Treatment

The HFNC was interrupted so the possible positive end-expiratory pressure caused by the device would be eliminated. Meanwhile, the patient briefly received a 15 L/min NRM. HFNC was restarted the next day as the patient was not tolerating the 15 L/min NRM. Moreover, from a physiological standpoint, it seemed improbable that the HFNC was the cause of the pneumomediastinum. No other interventions were taken.

## Outcome and follow-up

Oxygen was administered via HFNC over the next 7 days. During this period the FiO_2_ was gradually reduced, so that ultimately, plain nasal cannula was sufficient for oxygen delivery. After 9 days, the patient was discharged from the hospital without supplemental oxygen. After the diagnosis of pneumomediastinum and the restart of the HFNC, there were no signs of deterioration of the patient.

**Figure 5:**
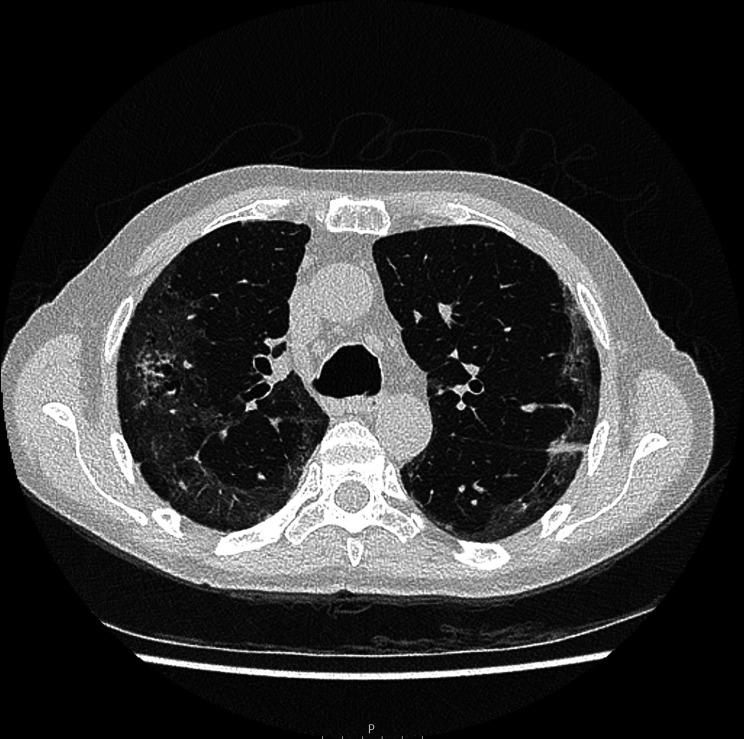
High-resolution CT of the chest (axial plane) at follow-up, showing no signs of pneumomediastinum.

A high-resolution CT of the chest was made 2 months after the first CT pulmonary angiogram. The pneumomediastinum had vanished completely (figure 5).

## Discussion

Pneumomediastinum can be divided into spontaneous pneumomediastinum, primarily caused by tobacco and recreational drug use, and secondary pneumomediastinum. The latter can be a result of traumatic or non-traumatic causes and iatrogenic causes. Common symptoms are dyspnoea, retrosternal chest pain and coughing. A pneumomediastinum usually occurs in younger patients. Mediastinal tissues of younger people are more flaccid and loose. It is thought that the air experiences more resistance due to the fibrosis of mediastinal sheats in older people. The diagnosis is confirmed via an X-ray of the chest, showing signs of radiolucent lines and bubbles in and around the mediastinum. In addition, a CT scan of the chest can evaluate the severity of the pneumomediastinum.^1^

HFNC is a device capable of providing patients with humidified oxygen at high rates of flow in various situations. It has proven to be a safe and useful additional therapy. Positive pressure ventilation via invasive mechanical ventilation is known to cause air leakage (eg, pneumomediastinum and pneumothorax). Since HFNC may generate a higher pressure (a positive end-expiratory pressure of 3–5 cm H_2_O is produced at flows of 30–50 L/min), it may potentially cause air leakage as well.^2 3^

In paediatric care setting, HFNC is a well-tolerated, non-invasive ventilation therapy. No adverse events have been described in the majority of the studies. A few cases, however, have been reported of paediatric patients who received HFNC suffering from air leakage.^4^ In adults, these adverse events have not been reported.^5^ Further literature search has not released extensive evidence of pneumomediastinum caused solely by HFNC. One case of a patient with pneumomediastinum in combination with subcutaneous emphysema, who was receiving supplemental oxygen therapy via HFNC has been reported. In this case, the pneumomediastinum was an incidental finding. Prior to the HFNC, the patient had received mechanical ventilation and suffered from acute respiratory distress syndrome. In this patient, the origin of the pneumomediastinum is seen as a result of acute respiratory distress syndrome.^6^ A few cases of pneumomediastinum in patients with COVID-19 have been reported. The probable mechanism in these patients was diffuse alveolar damage leading to cyst formation and subsequent rupture. In these patients, no other probable causes were detected.^7^

In our case, the HFNC treatment was interrupted briefly but was restarted shortly after. It is unlikely that HFNC was the cause of the pneumomediastinum in our case since HFNC is not able to generate large pressure differences at the alveolar level. Also, no previous reports were found in the literature. A recent case series in the UK showed that 1% of patients admitted with COVID-19 develop pneumothorax/pneumomediastinum. This can occur without pre-existing lung disease or mechanical ventilation.^8^ Although no HFNC use was mentioned in this study, 5% of the pneumothorax cases was on continuous positive airway pressure (CPAP) at that time. One case of pneumomediastinum was found to be related to the use of CPAP. However, CPAP may generate airway pressures that are four times higher than HFNC.

Therefore, the most probable cause for pneumomediastinum in our patient is diffuse alveolar damage due to COVID-19 pneumonia. Moreover, after restarting HFNC our patient remained stable. A CT scan, made 11 days after the first scan, showed a decrease of pneumomediastinum while still receiving oxygen via HFNC. In conclusion, HFNC does not seem contra-indicated while suffering from air leakage and it may even have clear advantages over CPAP or (non)-invasive ventilation.

**Patient’s perspective**

It was my first hospital admission. There were a lot of new impressions for me. The first few days of my hospital stay were a blur. I was very fatigued and was experiencing dyspnoea related to COVID-19. I was optimistic during my stay. I was not anxious for a possible deterioration of my situation. A situation that made quite an impression on me was that two relatively young patients were admitted to the Intensive Care due to progressive respiratory failure. I wondered whether I would be admitted to the intensive care unit (ICU) if my condition were to worsen, since I was much older than these patients. At that time there was a discussion in The Netherlands whether younger patients should get priority (concerning ICU admission) over older patients.

I did not experience the high-flow nasal cannula (HFNC) as unpleasant. After the restart of the HFNC, I noticed an improvement of my symptoms. However, 15 L/min non-rebreathing mask was unpleasant to me. I cannot remember if the restart of the HFNC had an effect on the subcutaneous emphysema.

**Learning points**

- Pneumomediastinum is a known complication of COVID-19.
- Supplemental oxygen therapy via high-flow nasal cannula (HFNC) is of great importance in the treatment of hypoxemia in patients with COVID-19.
- It is unknown whether HFNC may provoke or worsen air leakage in pneumomediastinum in adults.
- This case demonstrates that HFNC can be administered safely in COVID-19 patients with pneumomediastinum.
- The treatment of pneumomediastinum is supportive and the prognosis generally good.
